# Hypoxia inducible factor 1α-mediated LOX expression correlates with migration and invasion in epithelial ovarian cancer

**DOI:** 10.3892/ijo.2013.1878

**Published:** 2013-03-29

**Authors:** FANG JI, YOU WANG, LIHUA QIU, SHU LI, JING ZHU, ZHOU LIANG, YINSHENG WAN, WEN DI

**Affiliations:** 1 Department of Obstetrics and Gynecology, Renji Hospital, School of Medicine, Shanghai Jiao Tong University, Shanghai 200127;; 2 Shanghai Key Laboratory of Gynecologic Oncology, Focus Construction Subject of Shanghai Education Department, Shanghai 200127;; 3 Department of Obstetrics and Gynecology, Wuxi Maternal and Child Health Hospital, Nanjing Medical University, Wuxi 214000, P.R. China;; 4 Department of Biology, Providence College, Providence, RI 02918, USA

**Keywords:** lysyl oxidase, ovarian cancer, metastasis, hypoxia, hypoxia-inducible factor, reoxygenation

## Abstract

This study investigated the role of LOX in promoting invasion and metastasis of epithelial ovarian cancer in a hypoxic environment and its specific signal transduction pathway. Immunohistochemical detection of HIF-1α and LOX protein expression was performed on formalin-fixed paraffin sections of normal ovary, benign ovarian tumors, borderline and malignant epithelial ovarian tumor paraffin sample, using Mann-Whitney U test for independent comparisons and Wilcoxon signed-ranks test for paired comparisons. HIF-1α and LOX were knocked down in epithelial ovarian cancer cells (EOC), and HIF-1α/LOX regulation mechanism and LOX catalytic activity under hypoxia/reoxygenation microenvironment were explored. Cell migration and invasion ability in LOX inhibited HO8910 cells were investigated under hypoxia/reoxygenation conditions, using matrigel cell invasion and migration assays. We found that HIF-1α and LOX are highly expressed in epithelial ovarian cancer tissues, and the expression of both proteins is significantly correlated with the tumor grade, tumor diameter and lymph node metastasis. HIF-1α expression is positively correlated with the expression of LOX. Specifically, the expression of LOX and HIF-1α markedly increases under hypoxic conditions and decreases after reoxygenation. siRNA knockdown of LOX or β-aminoproprionitrile (βAPN), an inhibitor of LOX activity, that attenuates LOX activity, downregulates HIF-1α protein expression and inhibits HO8910 migratory and invasive abilities. LOX catalytic activity is significantly reduced under hypoxic conditions. Moreover, EOC cells display a marked increase in LOX-dependent FAK/AKT activation and cell migration following hypoxia/reoxygenation. Collectively, our study demonstrates that the hypoxia-HIF-1α, LOX-FAK/AKT pathway regulates the migration and invasion of epithelial ovarian cancer cells under hypoxia/reoxygenation conditions, thus, promoting metastasis of ovarian cancer.

## Introduction

Ovarian cancer is one of the most common gynecologic cancers, preceded only by cervical carcinoma. But epithelial ovarian carcinoma (or EOC) still ranks first as the leading cause of death among gynecological malignancies (1,2). Because of the broad transfer of abdominopelvic cavity, limitations of surgery and resistance or relapse after chemotherapy, the 5-year survival rate is only approximately 30% in 70% of patients with advanced disease (3).

Although significant progress has been made in understanding the biology of EOC, due to the asymptomatic nature of early ovarian cancer, patients are frequently diagnosed at advanced stages (III–IV) when intraperitoneal carcinomatosis is already apparent and the disease is disseminated (4). It is almost impossible to make an early detection (5), due to the lack of specificity of clinical symptoms and the absence of effective screening programs. Therefore, revealing the mechanisms of tumor invasion and metastasis and developing efficient targeted therapies may benefit the management of ovarian cancer.

During proliferation, invasion and migration of various tumors, cells may encounter hypoxia conditions, due to poor or altered vascularization (6). A number of studies have shown that hypoxia is an independent predictor of poor prognosis (7). A key regulator of the cellular response to oxygen deprivation is hypoxia-inducible factor-1 (or HIF-1), which comprises a constitutively expressed β-subunit and an oxygen-labile α-subunit (8). Hypoxic conditions stimulate HIF-1α to accumulate, which activates transcription of target genes involved in angiogenesis, energy metabolism, adaptive survival or apoptosis (9,10).

Lysyl oxidase (LOX) is a copper-dependent amine oxidase that is thought to function only in the extracellular milieu by cross-linking collagens or elastin to increase extracellular matrix tensile strength (11). This protein is essential for embryonic development, wound healing and adult tissue remodeling (12). The findings that LOX activity is modulated by oxygen levels, and also that LOX is able to regulate cell migration and adhesion (13), have generated considerable interest in the role of LOX during tumor progression. Both up- and downregulation of LOX have been observed in different cancer cell lines and primary tumors (12). However, in breast cancer, elevated LOX expression has been positively-correlated with migration and invasion (14). There is no report on the role of LOX in migration and invasion in ovarian carcinoma.

Hypoxia has been shown to upregulate LOX expression, via HIF-1α binding to hypoxia responsive elements in LOX promoter, leading to enhanced invasion in an invasive or meta-static breast cancer cell line. Conversely, inhibition of LOX expression and catalytic activity in this cell line significantly reduced the number of distant metastases to the lung and liver *in vivo* (15). Furthermore, overexpression of LOX in poorly invasive breast cancer cell lines results in an increase in *in vitro* migration and invasion (16). However, there is no research on the role of LOX in hypoxia of ovarian cancer. The aim of the present study was to investigate the expressions of LOX in ovarian cancer and relationships between expressions of LOX in hypoxia and clinical parameters or prognosis, and to explore the role of constitutive activation of LOX-HIF-1α signaling pathway in the invasion and metastasis of ovarian cancer. We hypothesized that hypoxia-induced LOX upregulates the expression of HIF-1α which promotes ovarian cancer cell invasion and metastasis.

We report the relationship between hypoxia/reoxygenation, LOX catalytic activity, and LOX-induced migration in the ovarian cancer cells HO8910 and HO8910-PM. We demonstrate that LOX expression correlates with HIF-1α in 61 cases ovarian tumor tissues and that hypoxia upregulates LOX and HIF-1α expression and migration/invasion of HO8910/HO8910-PM cells via HIF-1α and HIF-2α. Furthermore, the activation of AKT and MMPs/FAK is involved in LOX/HIF-1α-induced invasion of EOC cells. The identification of hypoxia-HIF-1α-LOX pathway provides novel insights into the mechanisms that control cancer cell migration in hypoxia and reoxygenation regions. Manipulation of the tumor microenvironment serves as a potential therapeutic approach for ovarian cancer.

## Materials and methods

### Sample preparation

Consecutive patients between February 2005 and August 2010 to Renji Hospital affiliated School of Medicine, Shanghai Jiao Tong University, who had histologically proven epithelial ovarian carcinoma (PEOC, n=41), borderline ovarian tumor (n=20), innocent ovarian tumor (n=27) and normal ovarian tissue (n=28) were studied. None of 116 patients had received radiation therapy or chemotherapy before surgery, and had no diabetes and other metabolic diseases. Their mean age was 56 and median age 60 years (range 28–76 years). A total of 41 PEOC patients had serious cystadenocarcinoma (n=25), mucinous cystadenocarcinoma (n=6), clear cell carcinoma (n=5) and endometrial carcinoma (n=5) by histological type. All the immunoreactions were separately evaluated by two senior pathologists.

### Immunohistochemical staining

Sections (4-*μ*m thick) were prepared from the paraffin-embedded tissues. Immunostaining was performed by the streptavidin-peroxidase (S-P) method (MaiXin, China). The specimens were incubated with primary antibody or negative control antibody, followed by biotinylated linking antibody and streptavidin peroxidase. The primary antibodies were anti-LOX (rabbit polyclonal antibody, 1:200, Novus, Littleton, CO, USA) and anti-HIF-1α (mouse monoclonal antibody, 1:400, BD Transduction Laboratories, San Jose, CA, USA). Breast cancer tissue sections with strong LOX and HIF-1α expression were used as positive controls. The primary anti-IgG was used as negative control. The peroxidase reaction was developed with DAB staining. LOX positive staining was found mainly in the cytoplasm, staining pale yellow-brown, small pieces or diffuse distribution. Staining intensity scoring criteria were: no color, 0; light yellow, 1; yellow, 2; and brown, 3. For the percentage tumor positivity, the following scoring was used: negative, 0; 1–25%, 1; 25–50%, 2; and >50%, 3. Both the staining intensity and percentage positivity scores were summed and tumors with scores ranging from 0 to 9 were assigned to: negative (0–1), + (2), ++ (3–4), +++ (≥5).

HIF-1α is present in the cytoplasm and nucleus. The appearance of brown particles in the nucleus or cytoplasm is considered as a positive immunohistochemistry score: no staining, 0; nucleus positive cells <10% and/or weak cytoplasmic staining, 1; the nucleus of 10–50% positive cells and/or moderate staining in the cytoplasm, 2; >50% positive cells in the nucleus and/or strong cytoplasmic staining, 3.

### Cells and culture conditions

Human ovarian carcinoma cell line HO8910-PM, HO8910 and SKOV3 were purchased from China Academy of Science Cell Bank (Shanghai, China). Human ovarian cancer line COC1 was purchased from China Typical Culture Collection Center (Wuhan, China). All cells were cultured in RPMI-1640 medium (Gibco-BRL, Grand Island, NY, USA) supplemented with 10% heat-inactivated fetal calf serum, 2 mM L-glutamine and 100 U/ml penicillin-streptomycin mixture (Gibco-BRL, Grand Island, NY, USA) in 5% CO_2_ and 95% air humidified atmosphere at 37°C. For experiments, cells were treated with the indicated concentrations of β-aminopropionitrile (βAPN; 100, 200 or 300 mM; Sigma Aldrich, St. Louis, MO, USA). For hypoxia, cells were cultured in a modular incubator chamber (Thermo Electron, Forma, MA, USA) that was infused with a mixture of 1% O_2_, 5% CO_2_ and 94% N_2_ at 37°C. Cells were incubated in normoxic or hypoxic condition for 4, 12 and 24 h, respectively.

### Quantitative PCR

Total RNA was isolated from cells in the logarithmic growth phase by TRIzol Reagent (Invitrogen, Carlsbad, CA, USA). First-strand cDNA synthesis was performed using the Superscript reverse transcription kit (Invitrogen, Germany). Q-PCR was carried out on an ABI Prism 7300 PCR Detection System (Applied Biosystems, Foster City, CA, USA) with fluorescence dye SYBR-Green (SYBR-Green Real-Time PCR Master mix, Toyobo, Japan). The sequences of the primers were as follows: LOX-F: 5′-TTGAGTCCTGGCTG TTATGATACC-3′, LOX-R: 5′-TGATGTCCTGTGTAGCGA ATGTC-3′. HIF-1α-F: 5′-ACTCAGGACACAGATTTAGA CTTG-3′, HIF-1α-R: 5′-TGGCATTAGCAGTAGGTTCTTG-3′. GAPDH-F: 5′-TGATGTCCTGTGTAGCGAATGTC-3′; GAPDH-R: 5′-TGATGTCCTGTGTAGCGAATGTC-3′. The thermal cycling conditions were: 95°C 60 sec, 40 cycles of 95°C for 15 sec, 60°C for 45 sec, Melting/Dissociation Curve Analysis. Data analysis was carried out by ABI sequence detection software using relative quantification. Relative expression levels were calculated using the 2^−ΔΔCt^ method. For quantification, the target sequence was normalized to the GAPDH mRNA levels.

### siRNA transfection

RNA interference and siRNA preparation was performed in 24 h, the following sequences for LOX and HIF-1α were used at 100 nM. HIF_1F: GCAUUGUAUGUG UGAAUUAdTdT; HIF_1R: UAAUUCACACAUACAAUGCdTdT; HIF_2F: CGAUGGAAGCACUAGACAAdTdT; HIF_2R: UUGUCUAGUGCUUCCAUCGdTdT; HIF_3F: CGAUCAGUUGUCACCAUUAdTdT; HIF_3R: UAAUGGUGACAACUGAUCGdTdT; LOX_1F: GGGCAGAUGUCAGAGAUUAdTdT; LOX_1R: UAAUCUCUGACAUCUGCCCdTdT; LOX_2F: GCACAGUUGUCAUCAACAUdTdT; LOX_2R: AUGUUGAUGACAACUGUGCdTdT; LOX_3F: GAAUCUGACUAUACCAACAdTdT; LOX_3R: UGUUGGUAUAGUCAGAUUCdTdT. Effects of siRNA for LOX (siLOX) or HIF-1α (siHIF-1α) were compared with those of a random siRNA sequence. Cells were plated onto 6-well plate and grown to 30–50% confluence before transfection. Transfection methods were: mixing 250 *μ*l serum-free Opti-MEM medium and 5 *μ*l liposomes, and mixing 250 *μ*l serum-free Opti-MEM medium and 5 *μ*l HIF-1α or LOX siRNA solution. Then all components were mixed for 5 min and incubated for 20 more min at room temperature. Twenty-four hours later, the cells were subjected to hypoxia treatment.

### Western blot analysis

The cells exposed to the various treatments were harvested, lysed and subjected to SDS-polyacrylamide gel electrophoresis and blotting, transferred to Immun-Blot membrane (polyvinylidene difluoride, Bio-Rad, Hercules, CA, USA), and immunoreactivity levels were evaluated by hybridization using the following antibodies: rabbit polyclonal antibody anti-LOX (1:1,000, Novus, Littleton, CO, USA); mouse monoclonal antibody (mAb) anti-HIF-1α (1:800, BD, USA); rabbit monoclonal antibody (mAb) β-actin antibody (1:2,000, Cell Signaling Technology, USA); FAK and Phospho-FAK (Tyr576) polyclonal antibody (1:1,000, Cell Signaling Technology, USA); AKT and Phospho-AKT (Ser473) polyclonal antibody (1:1,000, Cell Signaling Technology, USA); MMP-2 and MMP-9 polyclonal antibody (1:1,000, Cell Signaling Technology, USA). The signal was then detected by chemiluminescence with SuperSignal kit (Pierce, Rockford, IL, USA).

### Lysyl oxidase activity assay

Ovarian cancer cells were plated onto 96-well tissue culture plates (100 *μ*l/well) under different conditions, and stimulated as desired. Cell culture media were collected. LOX activity was measured using Lysyl Oxidase Activity Assay Kit (Fluorometric) (Abcam, UK). The assay reaction mixture consisted of HRP substrate, assay buffer, horseradish peroxidase and DMSO. Prior to assay, stock solutions and assay reaction mixture were prepared according to the protocols. A total of 50 *μ*l of assay reaction mixture was added to each well of the cell media, and those of lysyl oxidase standards. The mixture was incubated at 37°C for 10 to 30 min, protected from light. The fluorescence increase was measured with a fluorescence plate reader at Ex/Em = 530 to 570/590 to 600 nm (maximum Ex/Em = 540/590 nm).

### Matrigel cell invasion and migration assays

Ovarian cancer cells (5×10^4^) (48 h post-transfection or increasing concentrations of βAPN treated) were placed into the upper wells of a Matrigel-coated 24-well Transwell chamber (8-*μ*m pore size, 12-mm diameter, Costar, USA) in the 24-well plate and cultured in hypoxic or normoxic environment. The medium containing 20% FBS was added to the lower chamber. After 24 h of incubation, the non-invasive cells on the upper membrane surface were removed with a Q tip, and the invasive cells that invaded through the Matrigel and 8-mm pore size membrane were fixed with 4% paraformaldehyde and stained with crystal violet. The number of invasive cells was counted under inverted microscope (×200). Data presented are representative of four individual wells. Cell migration was assayed using a similar approach without Matrigel coating, and the treated cells in migration chamber were incubated under normoxic (N) or hypoxic (H) conditions for 16 h.

### Statistical analysis

Each experiment comparing the effects of different treatments used the same endometrial sample, and each experiment was repeated at least three times on different specimens. Data are presented as mean ± SD and analyzed by SPSS software using non-parametric statistical analysis (Mann-Whitney U test for independent comparisons and Wilcoxon signed-ranks test for paired comparisons). P≤0.05 was defined as statistically significant.

## Results

### Correlation between LOX/HIF-1α expression and clinico-pathological parameters in epithelial ovarian cancer

**Figure 1:**
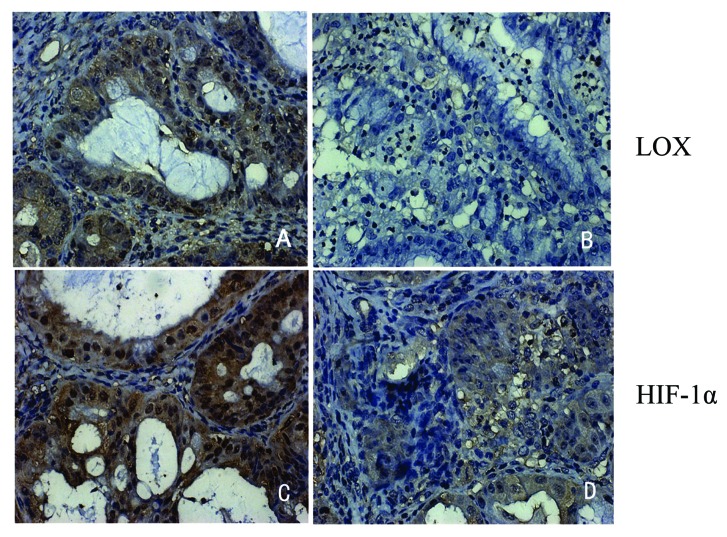
Immunohistochemical staining for LOX and HIF-1α in POEC. (A) Positive and (B) negative expression of LOX in POEC. (C) Strong and (D) weak positive expression of HIF-1α in POEC. Original magnification, ×200.

To determine whether LOX and HIF-1α expression increases in ovarian cancer tissues, we examined the expression of LOX and HIF-1α in 44 cases of human epithelial ovarian carcinoma, 20 cases of borderline ovarian tumor, 27 cases of benign ovarian tumor and 28 cases of normal ovarian tissues. Immunohistochemical data revealed that the predominant staining pattern of LOX and HIF-1α is cytoplasmic and/or nuclear (Fig. 1). LOX is positively expressed in 97.6% (40/41) epithelial ovarian cancer, 80% (16/20) borderline ovarian cancer, 48.1% (13/27) benign ovarian cancer and 7.1% (2/28) normal ovarian tissues (Table I). HIF-1α is positively expressed in 87.8% (36/41) epithelial cancer, 90% (18/20) borderline cancer, 40.7% (11/27) benign tumors and 17.9% (5/28) normal tissues (Table I). The data indicate that LOX and HIF-1α expression is related to ovarian cancer malignancy. The association of LOX and HIF-1α expression with clinical pathological parameters was then analyzed. Clinicopathological analysis showed that both LOX and HIF-1α expression are significantly associated with tumor FIGO classification (p=0.035 and p=0.032), tumor size (p=0.033 and p=0.032) and lymph node metastasis (p=0.016 and p=0.028, respectively) (Table II). Statistical analysis showed that LOX expression is correlated positively with the expression of HIF-1α (r=0.423, p=0.005) (Table III).

### Hypoxia promotes LOX and HIF-1α expression in epithelial ovarian cancer

**Figure 2:**
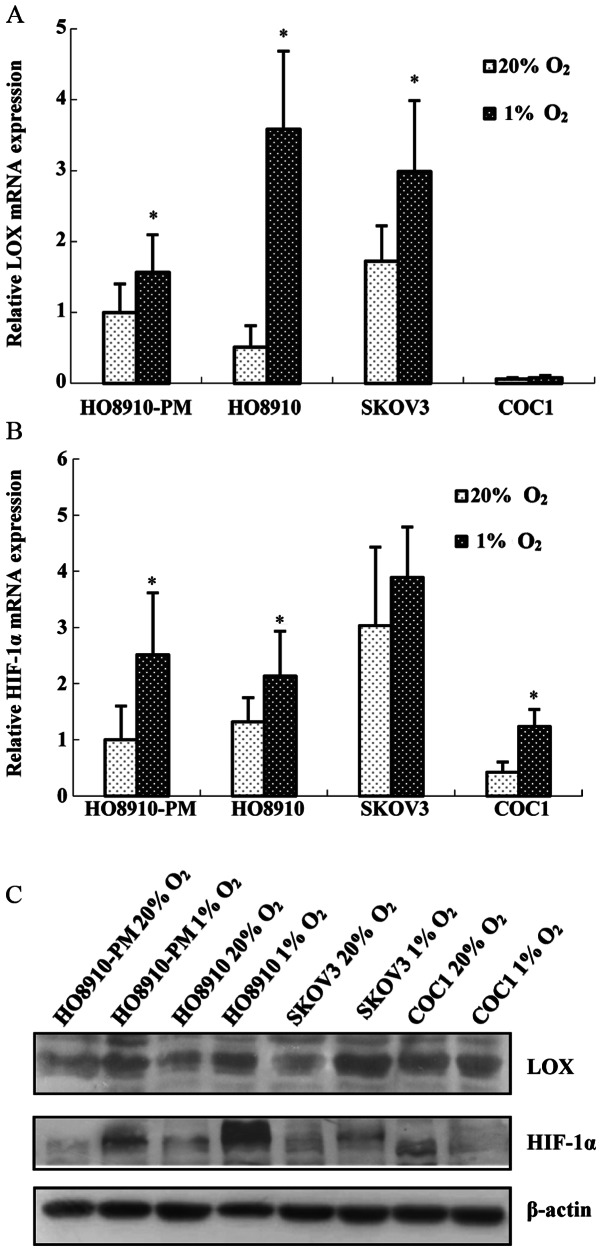
Hypoxia increases expression of LOX and HIF-1α in ovarian cancer cell lines. HO8910-PM, HO8910, SKOV3 and COC1 ovarian cancer cells were exposed to normoxia (20% O_2_) or hypoxia (1% O_2_) for 24 h. (A) Quantitative RT-PCR (Q-PCR) of *LOX* mRNA expression. (B) Q-PCR of *HIF-1α* mRNA expression; *GAPDH* was used as a reference gene (n=3 replicates per group); *P<0.05 vs. normoxic control cells. (C) Western blot analysis of LOX and HIF-1α protein expression; β-actin was used as a loading control.

**Figure 3:**
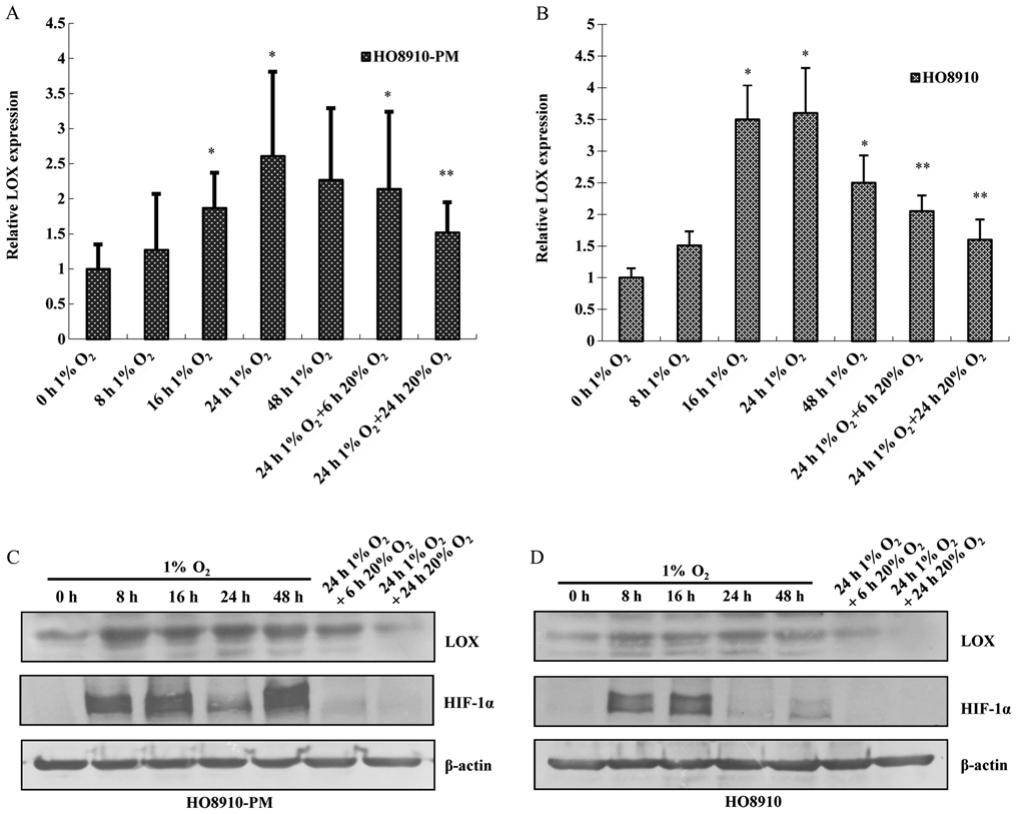
Hypoxia increases the expression of LOX and HIF-1α in ovarian cancer cells. Cells were cultured in hypoxia for 8, 16, 24 or 48 h, or hypoxia followed by reoxygenation, as indicated. Quantitative RT-PCR of *LOX* mRNA expression in (A) highly invasive/metastatic HO8910-PM ovarian cancer cells and (B) poorly invasive HO8910 ovarian cancer cells; the expression of *LOX* was normalized to *GAPDH* and expressed relative to cells cultured in 20% O_2_. Western blots of LOX and HIF-1α protein expression in (C) HO8910-PM and (D) HO8910 cells; β-actin was used as a loading control. ^*^P<0.05 vs. control cells cultured in 20% O_2_; **P<0.05 vs. cells cultured in 1% O_2_, ANOVA.

Ovarian cancer is a kind of multivessel solid tumor and hypoxia is accompanied with tumor growth. Under hypoxia condition, HIF acts as a considerable regulator which helps tumor cells to endure hypoxia and promote tumor infiltration and metastasis. Oxygen concentration <6% induces HIF-1α expression. To study how hypoxia affects LOX and HIF-1α expression, we exposed the ovarian cancer cell lines HO8910-PM, HO8910, COC1 and SKOV3 in 1% oxygen to mimic hypoxia condition. The results showed that all four cell lines are positive for LOX and HIF-1α mRNA. After being stimulated with 1% oxygen for 24 h, LOX and HIF-1α mRNA expression (Fig. 2A and B), as well as protein expression (Fig. 2C) increased significantly in HO8910-PM and HO8910 cells. However, LOX mRNA and protein expression increased marginally in COC1, SKOV3 cells (Fig. 2A and C). Therefore, we carried out hypoxia treatment in HO8910-PM (high invasion) and HO8910 (low invasion) cells. HO8910-PM, HO8910 cell lines were treated with 1% oxygen for different duration, and then given 20% oxygen for 6 or 24 h. Expression of LOX and HIF-1α was measured. The data showed that LOX mRNA and protein expression increases in a time-dependent manner under hypoxia condition with the peak at 24 h (Fig. 3A and B). HIF-1α protein expression also increases at 8 h after hypoxia treatment, peaks at 16 h, remains elevated at 24 h and returns to the basal level at 48 h. LOX along with HIF-1α mRNA and protein expression are downregulated due to reoxygenation administration for 6 h, reverting to the level before hypoxia treatment (Fig. 3C and D).

### Inter-regulation between LOX and HIF-1α in ovarian cancer cell lines under hypoxia condition

**Figure 4:**
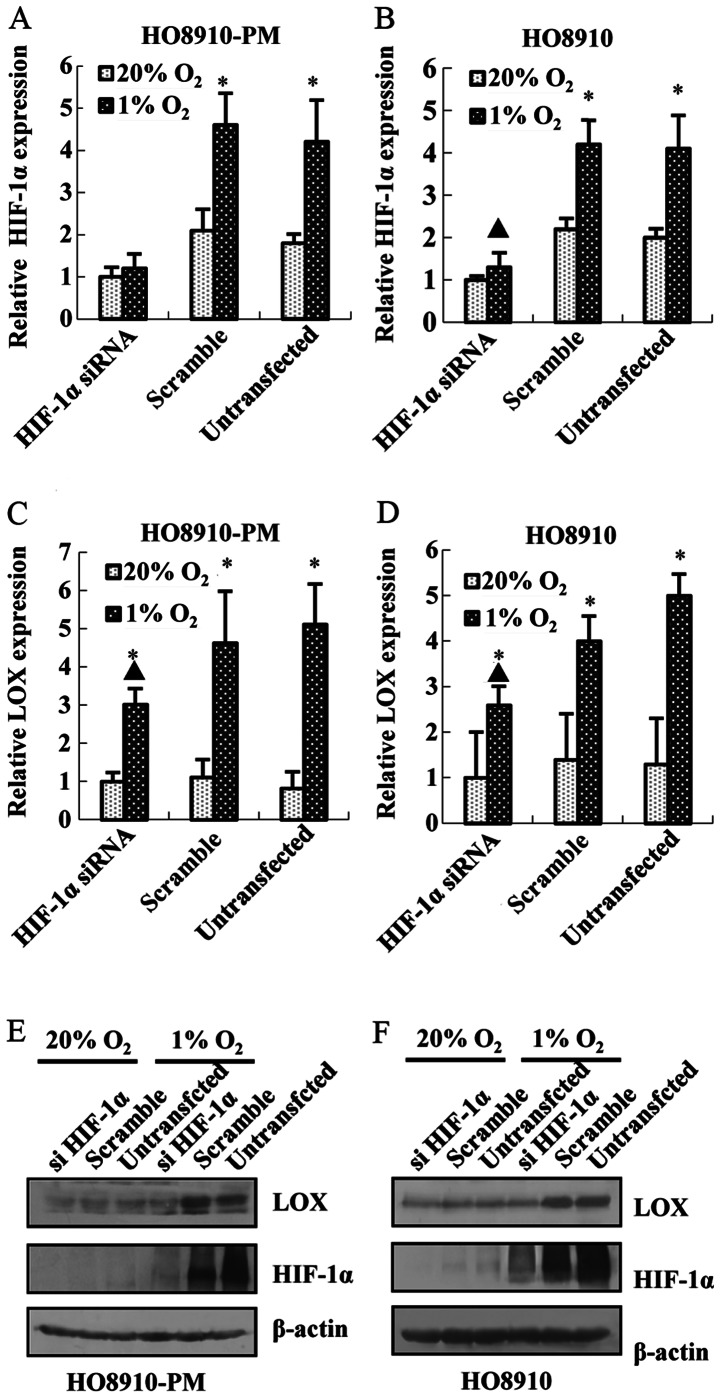
Knockdown of HIF-1α mRNA and protein expression by RNA interference in ovarian cancer cells. HO8910-PM and HO8910 cells were transfected with *HIF-1α*-siRNA or a scrambled siRNA, incubated for 24 h, and then exposed to normoxia (20% O_2_) or hypoxia (1% O_2_) for 24 h. Q-PCR analysis of *HIF-1α* mRNA expression in (A) HO8910-PM and (B) HO8910 cells. Q-PCR of *LOX* mRNA expression in (C) HO8910-PM and (D) HO8910 cells; *GAPDH* was used as a reference gene; ^▴^P<0.05 vs. control untransfected cells or non-specific siRNA-transfected cells under hypoxia, ^*^P<0.05 vs. 20% O_2_ control cells; ANOVA (n=3 replicates per group). Western blots of HIF-1α and LOX protein expression in (E) HO8910-PM and (F) HO8910 cells. Knockdown of HIF-1α expression using a *HIF-1α* siRNA simultaneously reduced LOX protein expression in cells cultured under hypoxic conditions; this effect was more obvious in HO8910 cells than HO8910-PM cells. Each experiment was performed at least three times.

**Figure 5:**
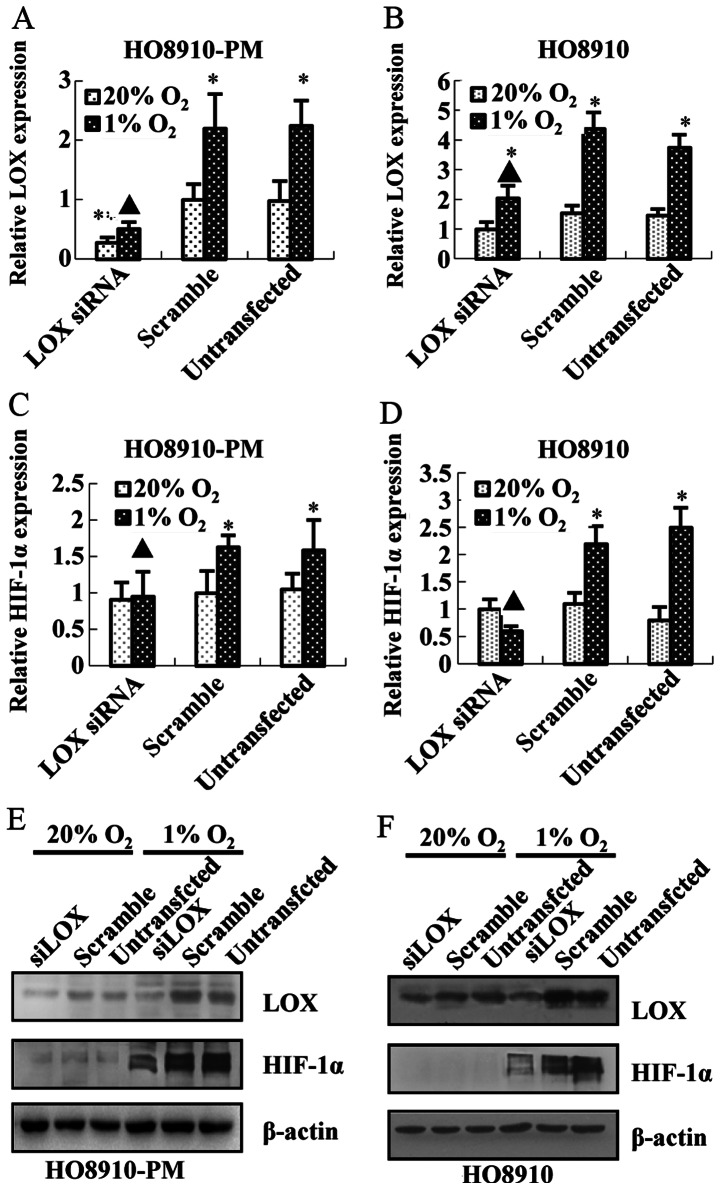
Effect of LOX on the regulation of HIF-1α expression in ovarian cancer cells cultured in normoxia or hypoxia. HO8910-PM and HO8910 cells were treated with siLOX, incubated for 24 h, and then exposed to normoxia (20% O_2_) or hypoxia (1% O_2_) for 24 h. Q-PCR of *LOX* mRNA expression in (A) HO8910-PM and (B) HO8910 cells. Q-PCR of *HIF-1α* mRNA expression in (C) HO8910-PM and (D) HO8910 cells; *GAPDH* was used as a reference gene; ^▴^P<0.05 vs. control untransfected cells or non-specific siRNA-transfected cells under hypoxia, ^*^P<0.05 vs. 20% O_2_ control cells; ANOVA (n=3 replicates per group). Western blots of HIF-1α and LOX protein expression in (E) HO8910-PM and (F) HO8910 cells. Knockdown of *LOX* mRNA expression (A and B) using siLOX reduced *HIF-1α* mRNA expression in hypoxic cells; this effect was more obvious in (D and F) HO8910 cells than (C and E) HO8910-PM cells. Each experiment was performed at least three times.

To study the relationship between LOX and HIF1α under hypoxia condition, HIF-1α siRNA was prepared and transfected in HO8910-PM and HO8910 cell lines. The results showed that HIF-1α siRNA transfection leads to the decrease of HIF-1α mRNA and protein expression in HO8910-PM (Fig. 4A and E) and HO8910 (Fig. 4B and F). Meanwhile, knockdown of HIF-1α down-regulates LOX mRNA (Fig. 4C and D) and protein expression (Fig. 4E and F). Furthermore, knockdown of LOX represses LOX mRNA and protein expression (Fig. 5A, B, E and F) as expected, and downregulates HIF-1α mRNA and protein expression (Fig. 5C–F) regardless of hypoxia. The data above suggest that LOX positively regulates HIF-1α expression and support the notion that LOX possibly acts as a pivotal upregulator on HIF-1α expression after hypoxia.

### LOX catalytic activity is necessary for regulating HIF-1α expression

**Figure 6:**
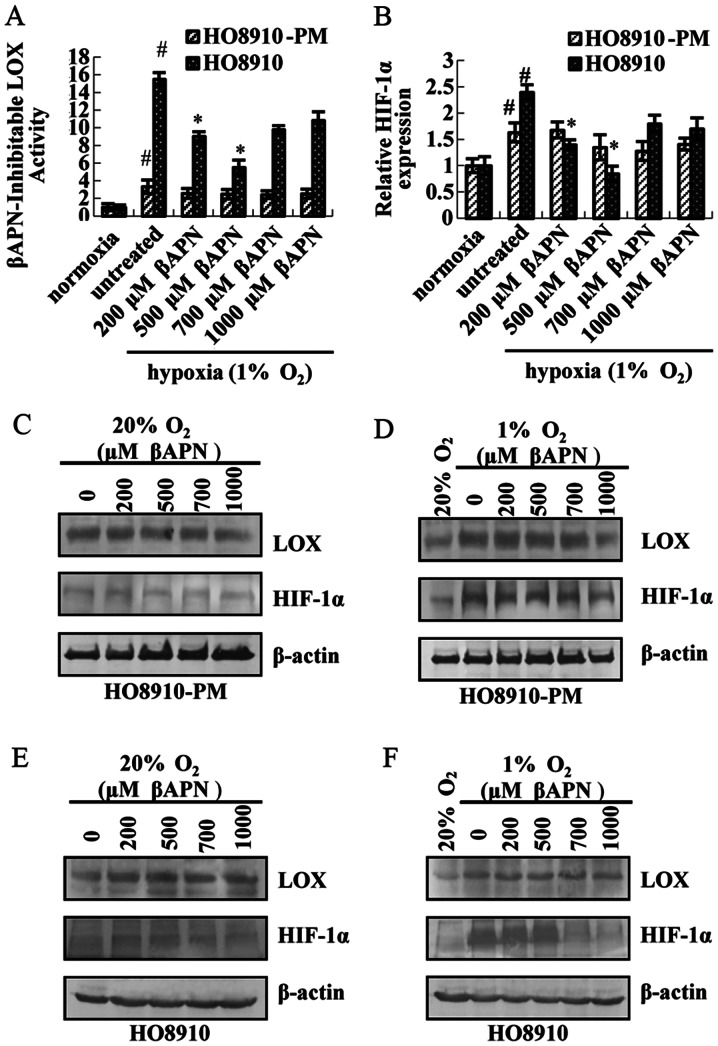
Effect of LOX catalytic activity on HIF-1α expression under hypoxic conditions. HO8910-PM and HO8910 cells were cultured under normoxia (20% O_2_), or hypoxia (1% O_2_) and exposed to different concentrations of βAPN (as indicated). (A) LOX activity (defined as βAPN-inhibitable hydrogen peroxide production) was measured using a commercial Lysyl Oxidase Activity Assay Kit. (B) Q-PCR analysis of *HIF-1α* mRNA expression; *GAPDH* was used as a reference gene. ^*^P<0.05 vs. normoxic control cells and ^#^P<0.05 vs. βAPN untreated hypoxic cells, ANOVA. Western blot analysis of LOX and HIF-1α protein expression in HO8910-PM cells cultured for 24 h in (C) 20% O_2_ or (D) 1% O_2_ in the absence or presence of increasing concentrations of βAPN. Western blot analysis of LOX and HIF-1α protein expression in HO8910 cells cultured for 24 h in (E) 20% O_2_ or (F) 1% O_2_ in the absence or presence of increasing concentrations of βAPN.

Recent studies have indicated that the biological functions of LOX proteins are dependent on its catalytic domain (11,17–19). We used the specific inhibitor of LOX catalytic activity, β-aminoproprionitrile (βAPN), to address the role of active LOX. The results showed that LOX catalytic activity (Fig. 6A), HIF-1α mRNA expression (Fig. 6B), LOX and HIF-1α protein expression (Fig. 6D) do not change dramatically in HO8910-PM cells under hypoxia condition after treatment by βAPN. LOX catalytic activity is inhibited by βAPN, especially at 500 *μ*M (Fig. 6A). LOX and HIF-1α mRNA (Fig. 6B), HIF-1α protein expression (Fig. 6E) is downregulated. In contrast, LOX protein expression remains unaltered. LOX and HIF-1α expression in both cells under normoxia condition are lower than that under hypoxia condition. Thus, no noticeable change exists in either LOX or HIF-1α at mRNA and protein level (Fig. 6C and E).

### LOX facilitated ovarian cancer cell migration and invasion depends on its catalytic activity

**Figure 7:**
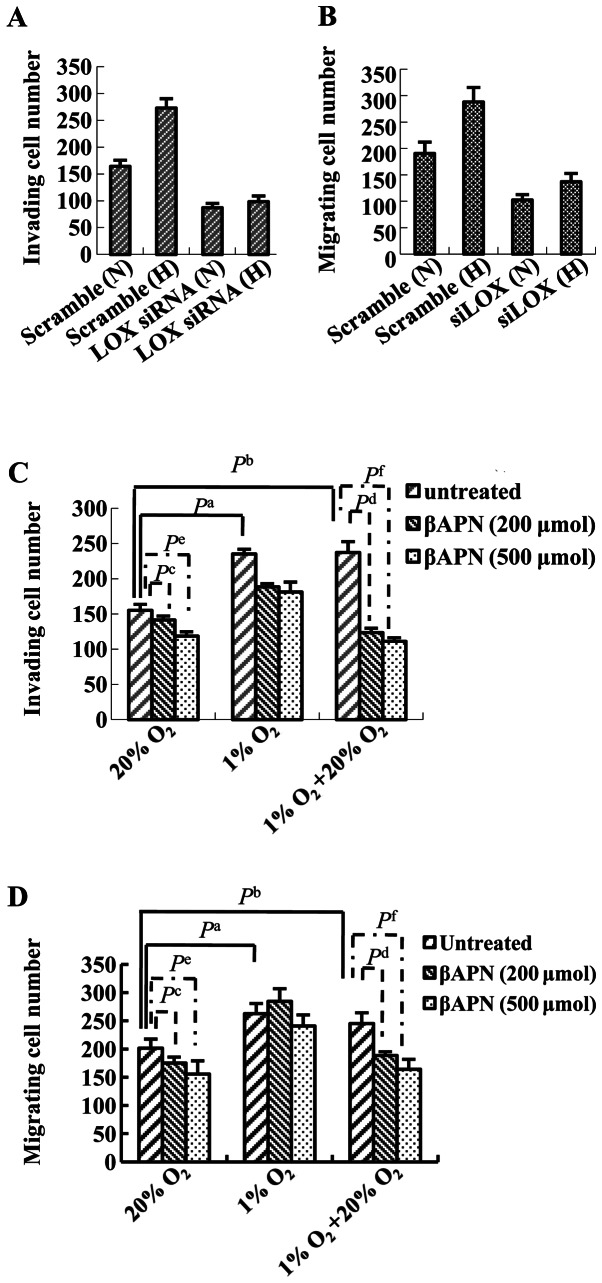
Effect of LOX on the invasion and migration of HO8910 cells under normoxic and hypoxic conditions. (A and B) HO8910 cells were transfected with scrambled siRNA or a siRNA against LOX and then subjected to the (A) Transwell invasion assay or (B) Transwell Matrigel migration assay under normoxic (N) or hypoxic (H) conditions for 24 h. Hypoxia increased cell invasion and migration; knockdown of *LOX* reduced cell invasion in both normoxic and hypoxic conditions. (C and D) HO8910 cells were cultured in 20% O_2_, 1% O_2_ or reoxygenation conditions (24 h at 1% O_2_ followed by 6 h at 20% O_2_). Where indicated, the cells were treated with increasing concentrations of βAPN for 24 h prior to and during the assays. (C) Transwell invasion assay. (D) Transwell Matrigel migration assay. A significant increase in cell invasion and migration were observed when HO8910 cells were cultured under reoxygenation or hypoxic conditions, compared to 20% O_2_. Inhibition of LOX catalytic activity using βAPN decreased cell invasion and migration in cells exposed to hypoxia; this effect was more obvious when the cells were cultured in 1% O_2_ followed by 6 h reoxygenation in 20% O_2_ (compared to 1% O_2_ or 20% O_2_ alone). Each experiment was performed at least three times and representative data are shown; data are the mean ± SD of five ×200 fields of view. N, normoxia; H, hypoxia. (A and B) Pa, Scramble (N) vs. Scramble (H); Pb, LOX siRNA (N) vs. Scramble (N); Pc, LOX siRNA (H) vs. Scramble (H). (C and D) Pa <0.05, untreated 1% O_2_ vs. untreated 20% O_2_; Pb <0.05, untreated 1% O_2_ + 20% O_2_ vs. untreated 20% O_2_; Pc <0.05, βAPN (200 *μ*mol) 20% O_2_ vs. untreated 20% O_2_; Pd <0.05, βAPN (200 *μ*mol) 1% O_2_ + 20% O_2_ vs. untreated 1% O_2_ + 20% O_2_; Pe <0.05, βAPN (500 *μ*mol) 20% O_2_ vs. untreated 20% O_2_; Pf <0.05, βAPN (500 *μ*mol) 1% O_2_ + 20% O_2_ vs. untreated 1% O_2_ + 20% O_2_.

Transwell migration assay and cell invasion assay, together with LOX siRNA transfection method were performed to study the effect of LOX on ovarian cancer cell migration and invasion capability. The results demonstrated that migration and invading capability in ovarian cancer cells increase under hypoxia condition as compared with those under normoxia condition without LOX siRNA transfection (Fig. 7A and B). Ovarian cancer cell migration and invasion capability decrease markedly after LOX siRNA transfection under both normoxia and hypoxia conditions (Fig. 7A and B). These data suggest that LOX is likely to be involved in ovarian cancer cell migration and invasion capability regulation. Ovarian cancer cell migration and invasion capability are blocked by βAPN in a dose-dependent manner under normoxia condition (Fig. 7C and D). βAPN impacts ovarian cancer cell migration and invasion capability to a lesser extent under hypoxia condition than that under normoxia condition (Fig. 7C and D). Reoxygenation followed by hypoxia rescues inhibitory activity of βAPN on ovarian cancer cell migration and invasion capability to the level under normoxia condition (Fig. 7C and D).

### LOX regulated ovarian cell migration and invasion via migration-related molecules and protein kinases

**Figure 8:**
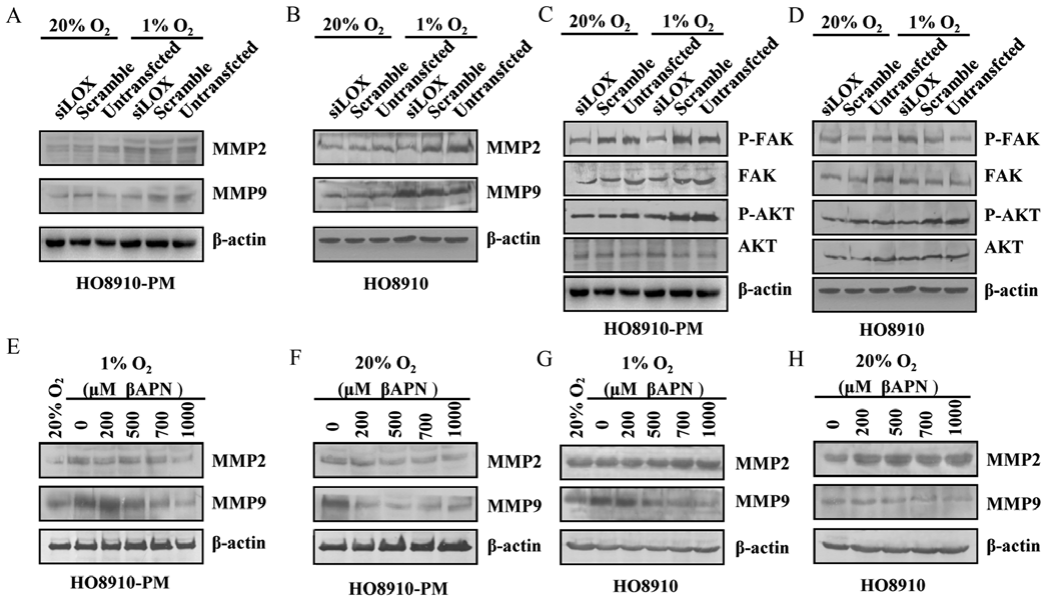
LOX catalytic activity regulates the expression of MMPs and activates the kinases FAK and Akt in ovarian cancer cells under normoxic or hypoxic conditions. HO8910-PM and HO8910 cells were transfected with *LOX*-siRNA or a scrambled siRNA for 24 h, and then exposed to normoxia (20% O_2_) or hypoxia (1% O_2_) for 24 h. (A and B) Western blot analysis of MMP2 and MMP9 expression. (C and D) Western blot analysis of total FAK, autophosphorylated FAK (Tyr576), total AKT and phospho-Akt (Ser473). (E and F) HO8910-PM and (G and H) HO8910 cells were cultured in 20% O_2_ for 24 h alone or under reoxygenation conditions (24 h at 1% O_2_ followed by 6 h 20% O_2_) in the absence or presence of increasing doses of βAPN, and then subjected to western blot analysis for MMP2 and MMP9.

To further study the mechanism through which LOX regulates ovarian cell migration and invasion, migration related molecules including MMP2, MMP9 and protein kinases which are involved in tumor migration such as FAK, AKT were examined after LOX siRNA transfection in HO8910-PM, HO8910 cells under normoxia and hypoxia conditions. The results showed that MMP9, FAK, AKT protein expression decreases in HO8910-PM cells under normoxia and hypoxia conditions (Fig. 8A and C). MMP2, P-FAK, P-AKT protein expression decreases in HO8910 cells under hypoxia condition (Fig. 8B and D). P-FAK, P-AKT protein expression decreases in HO8910 cells under normoxia condition (Fig. 8B and D). MMP2, MMP9 protein expression is downregulated by βAPN in HO8910-PM cells under reoxygenation condition followed by hypoxia similar to the level under normoxia condition (Fig. 8E and F). MMP9 expression is downregulated by βAPN in HO8910 cells under both hypoxia plus reoxygenation, and normoxia (Fig. 8G and H). Our data suggest that LOX likely regulates various types of ovarian cancer cells through different signaling pathways.

## Discussion

Epithelial ovarian cancer (EOC) is the leading cause of death from gynecologic cancer and the fifth most common cause of cancer mortality in women. EOC ranks first in gynecological cancer mortality and is prone to invasion and distant metastasis. In 2011, there was an estimated 21,990 new diagnoses and an estimated 15,460 deaths from this neoplasm in the United States, less than 40% of women with ovarian cancer are cured (20,21). The distant metastasis of EOC is one of the main reasons for their poor 5-year survival rate. Therefore, the current challenge is to understand the molecular mechanisms underlying epithelial ovarian cancer metastasis in order to design novel therapeutics.

Hypoxia is a characteristic of many malignancies arising from various sites (22). Ovarian cancer is a multi-blood vessel solid tumor. As tumor volume increases, there become a considerable number of hypoxic tumor cells. In hypoxic cancer cells, HIF-1α binds to the hypoxia responsive element (HRE) in the promoter region (13) of many target genes including LOX (15). LOX has been intensively studied in cancers as its importance in tumor progression becomes more thoroughly realized (16). Although LOX has been shown to play roles in tumor metastasis, the mechanism by which LOX expres sion is upregulated in ovarian cancer cells and the correlation with hypoxia are still poorly understood. Here, we present data that LOX is overexpressed in EOC tumor tissues compared with non-cancer tissues. Additionally, LOX is expressed intracellularily within ovarian cancer cells and facilitates cell migration through the regulation of HIF-1α. Furthermore, EOC cells display a marked increase in LOX-dependent FAK/AKT activation and cell migration following hypoxia/reoxygenation.

In this study, first, the association between hypoxia and increased LOX expression was validated in clinically relevant ovarian cancer paraffin-embedded tissues by immunohistochemical method. Using HIF-1α as a surrogate marker of hypoxia in malignant tissue, we observed a significant correlation between hypoxia and LOX expression. LOX and HIF-1α proteins are both overexpressed in ovarian cancer cells and the expression is significantly associated with advanced clinical stages and metastasis in EOC. Moreover, statistical analysis showed that high level of LOX correlates positively with the high expression of HIF-1α (r=0.423, p=0.005). Our data suggest that HIF-1α promotes EOC progression and metastasis via upregulation of LOX expression in EOC.

In order to further investigate whether hypoxia regulates LOX, we examined the effect of hypoxia on LOX expression in EOC cells. The expression of LOX mRNA and protein is strongly elevated in hypoxia-exposed HO8910 and HO8910-PM cells compared with the normoxic control cells. Furthermore, we demonstrated that the poorly invasive cancer cells HO8910, which express little to no endogenous LOX, aberrantly express high levels of LOX when exposed to hypoxic conditions. These findings are in agreement with those previously described in other cancer cells (15). Therefore, in addition to making invasive/metastatic cancer cells more invasive, hypoxia may induce LOX expression in poorly invasive cancer cells, thereby enabling them to acquire invasive competence.

It has been demonstrated that HIF-1α is a hypoxia responsive factor (8). We investigated whether hypoxia upregulates LOX via HIF-1α. Our data showed that the expressions of LOX mRNA and protein are greatly reduced when HIF-1α is downregulated by siRNA, suggesting that hypoxia may upregulate LOX via HIF-1α pathway in HO8910 and HO8910-PM cells. Studies have demonstrated that HIFs are involved in tumor invasion and metastasis (23). We investigated whether hypoxia could promote tumor invasion and migration via HIF-1α and LOX. In our study, we observed that the migration and invasion capability decreases markedly after LOX siRNA transfection in HO8910 cells under both normoxia and hypoxia conditions. Our data suggest that LOX is involved in ovarian cancer cell migration and invasion capability regulation and is related to HIFs. We further showed that LOX regulates cell motility/migration via LOX activity, supported by the data on βAPN, an inhibitor of LOX.

Multiple mechanisms may be involved in hypoxia-induced metastasis (13). Since hypoxia followed by reoxygenation may also provide a physiological pressure in tumors selecting for metastatic cell phenotypes (24,25), it is essential to determine whether hypoxia-induced LOX in ovarian cancer cells is active under hypoxic conditions. Our results show that under hypoxic conditions LOX is not catalytically active. This finding is in agreement with the LOX mechanism of action in that LOX requires oxygen in order to regenerate catalytic activity (11). As a consequence of its dependency on oxygen, hypoxia induced LOX is unable to induce cell migration until the microenvironment is reoxygenated. Moreover, this increase in migration is also correlated with the oxygen level and activation of MMPs and FAK. These novel findings validate the role of LOX in ovarian cancer metastasis and indicate the importance of reoxygenation when studying hypoxia induced phenomena.

While the invasive mechanism mediated by LOX-HIF-1α has been poorly understood in EOCs, accumulating data in other cancer cells have indicated that multiple signaling mechanisms exist to regulate cell migration. We showed that the LOX-HIF-1α mutual regulation mechanism activates the AKT pathway and thus promotes tumor cells migration. This result agrees with those published earlier showing that PI3K/AKT pathway increases the rate of HIF-1α protein synthesis (26). Such activation of AKT pathway by LOX is consistent with recently published results, which have shown that LOX modulates ovarian tumor progression by stimulating MMPs/FAK/AKT signaling. It has been shown that HIF-1α-inducible lysyl oxidase activates HIF-1α via PI3K/AKT pathway in colorectal cancer (26). PI3K and AKT pathways appear to operate independently (27), but sometimes the two pathways are intermingled (28). Further studies are needed to clarify the relationship between PI3K/AKT and other pathways in LOX signaling of ovarian cancer cells.

In conclusion, our study demonstrates that hypoxia-HIF-1α-LOX-AKT pathway regulates the invasion and migration of ovarian cancer cells under hypoxic/reoxygenation microenvironments. HIF-1α and LOX activation might play a role in promoting a more malignant phenotype of EOC and presents targets for novel therapeutic strategies.

## Figures and Tables

**Table I:** Expression of LOX and HIF-1α in PEOC tissue sections.

	No.	LOX	HIF-1α	P-value^a^
POEC	41	97.6% (40/41)	87.8% (36/41)	
Borderline ovarian tumor	20	80% (16/20)	90% (18/20)	P<0.001
Benign ovarian tumor	27	48.1% (13/27)	40.7% (11/27)	
Normal ovarian tissue	28	7.1% (2/28)	17.9% (5/28)	

a χ^2^ test of independence.

**Table II:** Correlation between the clinicopathological features and expression of LOX and HIF-1α in PEOC.

	LOX					HIF-1α				
	
Clinicopathological variable	−	+	++	+++	P-value	−	+	++	+++	P-value
Age (years)										
<60	0	4	8	8		3	6	4	7	
≥60	1	6	6	8	0.645	2	5	4	10	0.850
FIGO disease stage										
I–II	1	8	7	4		4	8	4	4	
III–IV	0	2	7	12	0.035^a^	1	3	4	13	0.032^a^
Tumor size (cm)										
≤2	1	8	6	4		4	8	2	5	
>2	0	2	8	12	0.033^a^	1	3	6	12	0.032^a^
Metastasis										
Positive	0	3	11	13		1	6	5	15	
Negative	1	7	3	3	0.016^a^	4	5	3	2	0.028^a^
Ascites										
Present	0	6	11	14		2	8	7	15	
Absent	1	4	3	2	0.126	3	3	1	3	0.191
CA125										
Abnormal	0	5	10	13		3	5	5	15	
Normal	1	5	4	3	0.172	2	6	3	2	0.109

a χ^2^ test of independence. FIGO, International Federation of Gynecology and Obstetrics.

**Table III:** Relationship between the expression of HIF-1α and LOX in PEOC.

		LOX			
HIF-1α	No.	-	+	++	+++
−	5	1	3	1	0
+	11	0	4	4	3
++	8	0	2	3	3
+++	17	0	1	7	9
Total	41	1	10	15	15

Expression of LOX correlated positively with the expression of HIF-1α (r=0.423, P=0.005).

## References

[1] Siegel R, Naishadham D, Jemal A (2012). Cancer statistics, 2012. CA Cancer J Clin.

[2] Jemal A, Siegel R, Ward E, Murray T, Xu J, Thun MJ (2007). Cancer statistics, 2007. CA Cancer J Clin.

[3] Rustin G, van der Burg M, Griffin C, Qian W, Swart AM (2011). Early versus delayed treatment of relapsed ovarian cancer. Lancet.

[4] Vergara D, Merlot B, Lucot JP, Collinet P, Vinatier D, Fournier I, Salzet M (2010). Epithelial-mesenchymal transition in ovarian cancer. Cancer Lett.

[5] Ricciardelli C, Oehler MK (2009). Diverse molecular pathways in ovarian cancer and their clinical significance. Maturitas.

[6] Choi JY, Jang YS, Min SY, Song JY (2011). Overexpression of MMP-9 and HIF-1α in breast cancer cells under hypoxic conditions. J Breast Cancer.

[7] Dayan F, Mazure NM, Brahimi-Horn MC, Pouyssegur J (2008). A dialogue between the hypoxia-inducible factor and the tumor microenvironment. Cancer Microenviron.

[8] Allen M, Louise Jones J (2011). Jekyll and Hyde: the role of the microenvironment on the progression of cancer. J Pathol.

[9] Semenza GL (2010). Defining the role of hypoxia-inducible factor 1 in cancer biology and therapeutics. Oncogene.

[10] Chen CL, Chu JS, Su WC, Huang SC, Lee WY (2010). Hypoxia and metabolic phenotypes during breast carcinogenesis: expression of HIF-1alpha, GLUT1, and CAIX. Virchows Arch.

[11] Lucero HA, Kagan HM (2006). Lysyl oxidase: an oxidative enzyme and effector of cell function. Cell Mol Life Sci.

[12] Payne SL, Hendrix MJ, Kirschmann DA (2007). Paradoxical roles for lysyl oxidases in cancer - a prospect. J Cell Biochem.

[13] Postovit LM, Abbott DE, Payne SL, Wheaton WW, Margaryan NV, Sullivan R, Jansen MK, Csiszar K, Hendrix MJ, Kirschmann DA (2008). Hypoxia/reoxygenation: a dynamic regulator of lysyl oxidase-facilitated breast cancer migration. J Cell Biochem.

[14] Nagaraja GM, Othman M, Fox BP, Alsaber R, Pellegrino CM, Zeng Y, Khanna R, Tamburini P, Swaroop A, Kandpal RP (2006). Gene expression signatures and biomarkers of noninvasive and invasive breast cancer cells: comprehensive profiles by representational difference analysis, microarrays and proteomics. Oncogene.

[15] Erler JT, Bennewith KL, Nicolau M, Dornhofer N, Kong C, Le QT, Chi JT, Jeffrey SS, Giaccia AJ (2006). Lysyl oxidase is essential for hypoxia-induced metastasis. Nature.

[16] Payne SL, Fogelgren B, Hess AR, Seftor EA, Wiley EL, Fong SF, Csiszar K, Hendrix MJ, Kirschmann DA (2005). Lysyl oxidase regulates breast cancer cell migration and adhesion through a hydrogen peroxide-mediated mechanism. Cancer Res.

[17] Polgar N, Fogelgren B, Shipley JM, Csiszar K (2007). Lysyl oxidase interacts with hormone placental lactogen and synergistically promotes breast epithelial cell proliferation and migration. J Biol Chem.

[18] Fogelgren B, Polgar N, Szauter KM, Ujfaludi Z, Laczko R, Fong KS, Csiszar K (2005). Cellular fibronectin binds to lysyl oxidase with high affinity and is critical for its proteolytic activation. J Biol Chem.

[19] Bouez C, Reynaud C, Noblesse E, Thepot A, Gleyzal C, Kanitakis J, Perrier E, Damour O, Sommer P (2006). The lysyl oxidase LOX is absent in basal and squamous cell carcinomas and its knockdown induces an invading phenotype in a skin equivalent model. Clin Cancer Res.

[20] Jemal A, Siegel R, Xu J, Ward E (2010). Cancer statistics, 2010. CA Cancer J Clin.

[21] Jemal A, Siegel R, Ward E, Hao Y, Xu J, Thun MJ (2009). Cancer statistics, 2009. CA Cancer J Clin.

[22] Cassavaugh J, Lounsbury KM (2011). Hypoxia-mediated biological control. J Cell Biochem.

[23] Gort EH, Groot AJ, van der Wall E, van Diest PJ, Vooijs MA (2008). Hypoxic regulation of metastasis via hypoxia-inducible factors. Curr Mol Med.

[24] Rofstad EK (2000). Microenvironment-induced cancer metastasis. Int J Radiat Biol.

[25] Ruan K, Song G, Ouyang G (2009). Role of hypoxia in the hallmarks of human cancer. J Cell Biochem.

[26] Pez F, Dayan F, Durivault J, Kaniewski B, Aimond G, Le Provost GS, Deux B, Clezardin P, Sommer P, Pouyssegur J, Reynaud C (2011). The HIF-1-inducible lysyl oxidase activates HIF-1 via the Akt pathway in a positive regulation loop and synergizes with HIF-1 in promoting tumor cell growth. Cancer Res.

[27] Denko NC, Fontana LA, Hudson KM, Sutphin PD, Raychaudhuri S, Altman R, Giaccia AJ (2003). Investigating hypoxic tumor physiology through gene expression patterns. Oncogene.

[28] Choi YK, Kim CK, Lee H, Jeoung D, Ha KS, Kwon YG, Kim KW, Kim YM (2010). Carbon monoxide promotes VEGF expression by increasing HIF-1alpha protein level via two distinct mechanisms, translational activation and stabilization of HIF-1alpha protein. J Biol Chem.

